# Sequential use of fractional 10600 nm CO_2_ and 1540 nm laser for the treatment of labia majora: a minimally invasive synergistic approach to vulval rejuvenation

**DOI:** 10.1097/JW9.0000000000000265

**Published:** 2026-06-25

**Authors:** Catarina Capela Herz, Laura Pieri, Irene Fusco

**Affiliations:** a Clinica dermatologica Dra Catarina Capela, São Paulo-Brazil; b El.En. Group, Department of Clinical Research and Practice, Calenzano, Italy.

**Keywords:** fractional 10.600 and 1540 nm lasers, labia majora rejuvenation, minimally invasive technique

**What is known about this subject in regard to women and their families?**

- The aging of the labia majora (the external genital labia) is a natural process that can be influenced by various factors, including age, hormonal fluctuations, genetics, and lifestyle. Over time, the labia majora may undergo changes that alter their shape, size, and elasticity. Labia majora can be rejuvenated using a variety of techniques, including radiofrequency inductions, platelet-rich plasma injection, lipofilling (autologous fat transplant), hyaluronic acid filler injection, and surgery to restore volume and improve appearance. Many women seek rejuvenation to improve the appearance of the labia majora, especially if they feel their genital area looks aged or lacks fullness (aesthetic purposes). Some women experience discomfort or loss of sensation due to changes in the labia majora, such as sagging or a decrease in volume, which can affect sexual satisfaction or cause irritation during daily activities (functional impairment). Nonsurgical treatment is a valid alternative to labial surgery, using energy-based devices, including radiofrequency and laser (CO_2_, erbium:yttrium aluminium garnet) to treat various skin concerns in the genital area, including labial tissue. CO_2_ laser is typically used for vaginal rejuvenation to improve skin texture, vaginal tightness, elasticity, and vaginal mucosa by stimulating collagen production.

**What is new from this article as messages for women and their families?**

- The laser system used in this study, which combines 2 wavelengths (CO_2_ and 1540 nm), one more ablative and the other more thermal, is able to create a synergy that amplifies aesthetical benefits for the labia majora rejuvenation. Thanks to its deep and homogeneous thermal stimulation, the synergy of the 2 wavelengths promotes increased cell turnover for faster healing, much benefitting the patient’s recovery time. The special sequential emission also synergistically enhances tissue shrinkage with collagen production to amplify the remodeling effect of lax tissues to increase their tone.

*Dear Editor,*

To rejuvenate the labia majora, various techniques can be used, including radiofrequency treatments, platelet-rich plasma injections, lipofilling (fat transfer), hyaluronic acid filler injections, and surgery.^1^

Nonsurgical labial treatments, using radiofrequency and lasers (CO_2_, erbium:yttrium aluminium garnet), offer an alternative to surgery for various genital skin concerns.^2^

CO_2_ laser vaginal rejuvenation improves texture, tightness, elasticity, addressing dryness, laxity, and labial appearance with minimal downtime.^2^

It is also a safe labiaplasty tool for functional and cosmetic purposes.^3^

Combining a CO_2_ laser with a 1540 nm wavelength enhances skin remodeling, minimizing discomfort and downtime.^4^ The 1540 nm wavelength promotes fibroblast activity and collagen synthesis.^5^

This study evaluated the safety and effectiveness of a sequential CO_2_ and 1540 nm laser for labia majora rejuvenation.

A total of 9 women with a mean age of 54 ± 5 years (50-65 years) and with Fitzpatrick skin phototypes between II and IV (33% patients presented phototype II, 44% patients presented phototype III, and 22% patients presented phototype IV), were enrolled in this study. All the patients requested the treatment for aesthetic reasons, and 33% of them also for functional ones.

Patients underwent an average of 1.4 ± 0.5 treatment sessions every 2-3 months, with a dual-wavelength system (DuoGlide, DEKA M.e.l.a, Florence, Italy), equipped with µ-Scan V^2^LR scanning system with a vulvar straight terminal, that includes a 10,600 nm CO_2_ laser device (60 W) and a 1540 nm diode laser (10 W). The following settings were applied: for the 10,600 nm wavelength, power of 5-7 W, type of pulse H-pulse, spacing of 500-800 µm, and stack 2; whereas for the 1540 nm wavelength, power 3 W, and dwell time 5 ms.

A photographic evaluation was used to assess the aesthetic effects of treatment on the labia majora. The 5-point Global Assessment of Improvement Scale rating overall improvement (excellent, good, moderate, slight, or no improvement); both patient and physician evaluations were included. Participants were monitored for treatment-related issues or side effects (none, mild, moderate, or severe) and used the visual analog scale to measure pain (no pain, mild pain, moderate pain, severe pain, and worst possible pain).

Aesthetic outcomes were assessed by comparing postlaser treatment photos to the baseline (Fig. 1). At trial’s end, patient assessment (44% excellent, 45% good, and 11% moderate) and physician assessment (44% excellent and 56% good) showed similar results. Combining both assessments offers a balanced view of progress.

**Fig. 1.**
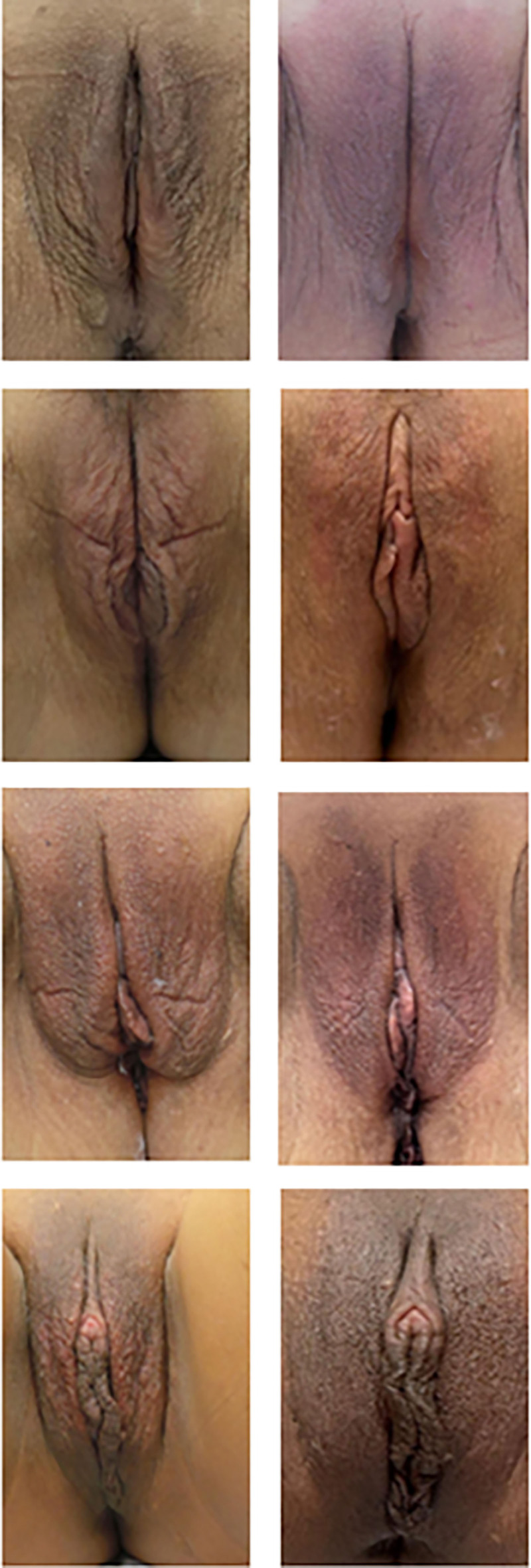
Aesthetic photographic examination of some different clinical cases of labia majora. Left panels show the view of the patient’s labia majora before the laser therapy. Right panels show the view of patient’s labia majora at 3 months follow-up post last laser treatment session.

Patients were monitored for adverse effects: 33% had no erythema, and 67% had mild erythema; 56% had no edema, and 44% had mild edema. Only 1 patient experienced itching, which resolved in hours. Average discomfort/pain, measured using the visual analog scale, was 0.5 ± 1. All results are shown in Table 1.

**Table 1:** Clinical outcomes and adverse events

Category	Parameter	Results
GAIS—patient assessment	Excellent	44% of patients
	Good	45% of patients
	Moderate	11% of patients
	Slight	0% of patients
	No improvement	0% of patients
GAIS—physician assessment	Excellent	44% of patients
	Good	56% of patients
	Moderate	0% of patients
	Slight	0% of patients
	No improvement	0% of patients
Erythema	None	33% of patients
	Mild	67% of patients
Edema	None	56% of patients
	Mild	44% of patients
Itching	Present	1 case (resolved within hours)
	Absent	Remaining patients
Pain (VAS)	Mean ± SD	0.5 ± 1

GAIS, Global Assessment of Improvement Scale; VAS, Visual Analog Scale.

This study showed significant improvement in patient and physician scores after laser treatment, with visual enhancement contributing to satisfaction.

The study system combines CO_2_ and 1540 nm wavelengths for labia majora rejuvenation, offering enhanced aesthetic benefits. This synergy promotes faster healing, tissue shrinkage, and increased collagen production, improving skin tone and tightening lax tissues.^4,5^ Although additional investigation with larger samples is required to corroborate these findings, the study device may represent a safe and effective therapy for labia majora rejuvenation.

## Conflicts of interest

The authors made the following disclosures: I.F. and L.P. are employed at El.En. Group. The other authors have no conflicts of interest to declare.

## Funding

None.

## Study approval

N/A

## Author contributions

CCH, LP, IF: Conception and design, collection and assembly of data, and data analysis and interpretation. CCH: Administrative support and provision of study materials or patients. All authors: Manuscript writing and final approval of the manuscript.

## Patients consent

Written informed consent was obtained from the patients for the publication of this case series and accompanying images. A copy of the written consent is available for review by the editorial office of this journal.

## Ethical Statement

The authors are accountable for all aspects of the work in ensuring that questions related to the accuracy or integrity of any part of the work are appropriately investigated and resolved. Ethical approval was not required as the study device is already CE marked since 14/04/2021. No activity was carried out outside the scope of the device’s intended purpose or that no additional invasive or burdensome procedures were carried out compared with procedures performed under the normal condition of use of the device.
